# A Systematic Analysis of miRNA Transcriptome in Marek’s Disease Virus-Induced Lymphoma Reveals Novel and Differentially Expressed miRNAs

**DOI:** 10.1371/journal.pone.0051003

**Published:** 2012-11-30

**Authors:** Ling Lian, Lujiang Qu, Yanmei Chen, Susan J. Lamont, Ning Yang

**Affiliations:** 1 Department of Animal Genetics and Breeding, National Engineering Laboratory for Animal Breeding, MOA Laboratory of Animal Genetics and Breeding, College of Animal Science and Technology, China Agricultural University, Beijing, China; 2 Department of Animal Science, Iowa State University, Ames, Iowa, United States of America; The Roslin Institute, University of Edinburgh, United Kingdom

## Abstract

Marek’s disease is a lymphoproliferative neoplastic disease of the chicken, which poses a serious threat to poultry health. Marek’s disease virus (MDV)-induced T-cell lymphoma is also an excellent biomedical model for neoplasia research. Recently, miRNAs have been demonstrated to play crucial roles in mediating neoplastic transformation. To investigate host miRNA expression profiles in the tumor transformation phase of MDV infection, we performed deep sequencing in two MDV-infected samples (tumorous spleen and MD lymphoma from liver), and two non-infected controls (non-infected spleen and lymphocytes). In total, 187 and 16 known miRNAs were identified in chicken and MDV, respectively, and 17 novel chicken miRNAs were further confirmed by qPCR. We identified 28 down-regulated miRNAs and 11 up-regulated miRNAs in MDV-infected samples by bioinformatic analysis. Of nine further tested by qPCR, seven were verified. The gga-miR-181a, gga-miR-26a, gga-miR-221, gga-miR-222, gga-miR-199*, and gga-miR-140* were down-regulated, and gga-miR-146c was up-regulated in MDV-infected tumorous spleens and MD lymphomas. In addition, 189 putative target genes for seven differentially expressed miRNAs were predicted. The luciferase reporter gene assay showed interactions of gga-miR-181a with *MYBL1*, gga-miR-181a with *IGF2BP3*, and gga-miR-26a with *EIF3A*. Differential expression of miRNAs and the predicted targets strongly suggest that they contribute to MDV-induced lymphomagenesis.

## Introduction

The miRNAs are small noncoding RNAs that act as a novel species of gene regulators, playing important roles in multiple biological processes, including growth, development, and differentiation [1], [2]. Generally, miRNAs negatively modulate gene expression by reducing mRNA stability and interfering with translation [3], [4]. In addition to reports on the direct role of miRNAs on protein-encoding genes, there are increasing numbers of recent studies on miRNA and cancer. Many miRNAs are aberrantly expressed in cancers. Some miRNAs act as oncogenes, while others show tumor suppressor activities [5], [6]. miRNAs mediate neoplastic transformation [7], and are strongly linked to lymphoma development in hematologic malignancies [8]–[10]. The profiling of miRNA expression is a useful tool for classifying various types of tumors by developmental lineage and differentiation state [11]. Recently, miRNAs were reported to be involved in virus-induced, in addition to non-infectious, forms of cancer [12], [13]. Oncogenic viruses have been implicated in a large proportion of neoplasms in animals, and they play key roles in molecular pathways of neoplastic transformation.

Marek’s disease (MD) is a highly contagious, lymphotropic, neoplastic disease. Although vaccines have been used since 1972, MD remains a serious threat to poultry health due to the increasing virulence of MDV under the pressure of vaccines [14]. Marek’s disease virus (MDV) induced MD lymphoma, a natural and rapid-onset T-cell lymphoma, is also an excellent biomedical model for virus-induced lymphoma [15], [16]. Recently, studies on host and virus miRNAs in the MD model system have been reported [17]–[23]. Burnside et al. (2008) identified miRNAs in MDV-infected chicken embryo fibroblasts (CEF) using deep sequencing technology [18]. In their study, 125463 high quality reads showed exact matches to the chicken genome, and 63 novel miRNA candidates were identified. Some host miRNAs, including gga-miR-let-7, gga-miR-199a-1, gga-miR-26a, gga-miR-181a, and gga-miR-16, were expressed at lower levels in MDV-induced tumors than non-infected spleens, indicating their potential importance in tumorigenesis. Yao et al. [21] investigated miRNA expression profiles in seven MDV-transformed cell lines by using microarray. Their study showed that several host-encoded miRNAs, including gga-miR-155, gga-miR-150, gga-miR-451, gga-miR-26a, and gga-miR-223, were down-regulated in all MDV-transformed cell lines compared to normal splenocytes, and nine MDV-1-encoded miRNAs were up-regulated in all of the MDV-transformed cell lines compared to the MDV-negative reticuloendotheliosis virus (REV)-T-transformed cell line AVOL-1.

Previous studies have mainly focused on miRNA profiles of MDV-infected CEF and MDV-transformed cell lines and expression of several individual miRNAs in MDV-induced splenic tumors. To date, there have been no reports of comprehensive miRNA expression profiles in lymphoma induced by MDV. Deep sequencing technology, with its high sensitivity, is highly suited for small RNA discovery [18],[24]. In the current study, we performed Solexa deep sequencing of MDV-infected and non-infected samples, including MDV-infected whole spleen (tumorous spleen) containing both tumor and non-tumor tissue, lymphoma isolated from MDV-infected liver, non-infected spleen, and non-infected peripheral blood lymphocytes, to elucidate repertoires of host and virus miRNAs. Our objective was to characterize miRNA involvement in pathophysiological processes of tumor transformation.

## Results

### Overview for Deep Sequencing

**Figure 1:**
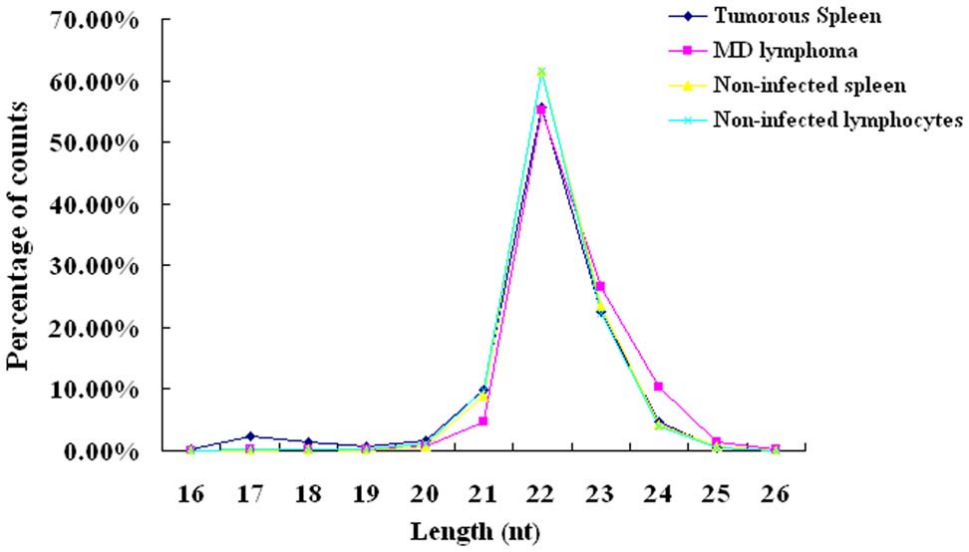
Size distribution of known miRNAs and novel miRNA candidates.

Raw data was processed as described in the Materials and Methods section. The overview of distribution of count percentage and numbers, from raw data to cleaned sequences, is shown in Table 1. MDV-infected tumorous spleen and MDV-induced lymphoma from liver were enriched in mRNA, RFam, and Repbase (13.88% and 12.81% by counts, respectively) compared to non-infected spleen and lymphocytes (2.61% and 2.12% by counts, respectively). Based upon the alignment results (see Materials and Methods for details), sequences were classified into known miRNAs and three groups of novel miRNA candidates. Subgroup1, which we considered novel 5p- or 3p-derived miRNA candidates, contains sequences mapped to the 5p or 3p arm of the pre-miRNA hairpin opposite to the annotated mature miRNA-containing arm. Subgroup2 includes miRNA candidates mapped to vertebrate or herpesvirus (except chicken or MDV) precursor miRNAs. Subgroup3 includes miRNA candidates not mapped to vertebrate or herpesvirus miRNA precursor, but mapped to the chicken or MDV genomes; and the extended genome sequences forming hairpins. The number of known miRNAs and novel miRNA candidates is listed in Table 2. The size distribution was analyzed and was not significantly different in the four libraries. The majority of the reads were 21–23 nt, with 22 nt as the most frequent size (Figure 1).

**Table 1:** Overview of reads from raw data to cleaned sequences.

		MDV-infected				Non-infected			
RNA class	% average	Tumorous spleen		Lymphoma(liver tumor)		Spleen		Lymphocytes(peripheral blood)	
		# counts	%	# counts	%	# counts	%	# counts	%
Raw data	\	7,764,498	100%	6,356,397	100%	6,362,545	100%	7,596,330	100%
Adapter dimer filter^a^	0.48	76,808	0.99	42,924	0.68	3,715	0.06	15,123	0.2
Junk filter^b^	1.01	26,945	0.35	23,507	0.37	196,229	3.08	16,743	0.22
Length filter^c^	5.98	801,493	10.32	460,394	7.24	238,932	3.76	196,453	2.59
Simple sequence filter^d^	0.07	8,646	0.11	5,976	0.09	1,840	0.03	3,708	0.05
Copy#<3	5.48	719,700	9.27	346,675	5.45	198,874	3.13	308,433	4.06
mRNA,RFam,Repbase^e^	7.86	1,077,754	13.88	814,183	12.81	165,784	2.61	161,361	2.12
mRNA^f^	0.22	39,164	0.50	16,057	0.25	2,783	0.04	5,189	0.07
rRNA^g^	2.90	390,752	5.03	317,444	4.99	76,257	1.20	28,290	0.37
tRNA^g^	0.73	102,413	1.32	54,516	0.86	13,871	0.22	41,056	0.54
snRNA^g^	0.54	48,209	0.62	93,853	1.48	2,003	0.03	2,205	0.03
snoRNA^g^	0.17	22,641	0.29	9,178	0.14	2,936	0.05	13,480	0.18
Repeats^h^	5.34	799,335	10.29	495,391	7.79	117,624	1.85	106,753	1.41
Cleaned sequences	79.14	5,053,152	65.1	4,662,738	73.4	5,557,171	87.34	6,894,509	90.76

a Adapter dimer: 5′ ADT, 3′ ADT & 5′ ADT and 3′ADT joined together without insertion.

b Junk sequence: 80% of A, or C, or G, or T, or 3N; 10 repeats of dimer, 6 repeats of trimer, or 5 repeats of tetramer; 7 consecutive A, 8 consecutive C, 6 consecutive G or 7 consecutive T; contained only A and C or only G and T.

c Sequences with length <16 nt or >26 nt were removed.

d Simple sequence: sequence with more than 80% of A, or C, or G, or T, or 3N in it.

e Sequences passing mRNA, RFam, Repbase filter.

f mRNA: ftp://hgdownload.cse.ucsc.edu/goldenPath/galGal2/bigZips/.

g Rfam(V9.1): ftp://ftp.sanger.ac.uk/pub/databases/Rfam/9.1/.

h repeat-repbase (V15.13): http://www.girinst.org/repbase/update/index.html.

Note: There is overlap in mapping of reads with mRNA, rRNA, tRNA, snRNA, snoRNA and repeats.

**Table 2:** Number of known chicken miRNAs and novel miRNA candidates in four samples.

	MDV infected		Non-infected	
Group	Tumorous spleen	Lymphoma(liver tumor)	Spleen	Lymphocytes (peripheral blood)
Known miRNAs	160	138	143	159
Novel miRNA candidates in subgroup1	44	38	42	61
Novel miRNA candidates in subgroup2	96	86	81	113
Novel miRNA candidates in subgroup3	498	142	71	231

### isomiRs of Known miRNAs and the Expression Patterns of miRNA and miRNA*s

In total, 187 known chicken miRNAs were identified. Among them, 165 were in MDV-infected samples, 174 were in non-infected samples, and 117 were identified in all four samples. Three miRNAs (gga-miR-135a, gga-miR-375, and gga-miR-429) were uniquely detected in MDV-infected samples, and three miRNAs, (gga-miR-1728-3p, gga-miR-1729*, and gga-miR-3530) were uniquely detected in non-infected samples. Additionally, 16 known MDV miRNAs were detected in both tumorous spleen and MD lymphoma from liver. Identified known chicken and MDV miRNAs are listed in Table S1. As previously reported [24]–[26], the present study identified many variants for known miRNAs, especially at the 3′ and 5′ends. These variants were defined as “isomiRs”, which frequently had higher counts than the corresponding known reference miRNA sequences listed in miRBase. The most abundant sequence in alignment with known chicken miRNAs listed in miRBase 16.0 was identified as a representative of the alignment and designated the “dominant isomiR”. The dominant isomiR was further defined based upon its differences from the reference miRNA sequence in miRBase 16.0. For example, gga-miR-1306_L-3R+2 (L = 5′end; R = 3′end) is a variant of gga-miR-1306 and is 3 nt shorter at 5′ end and 2 nt longer at 3′ end than gga-miR-1306. gga-miR-1579_21GA contains a variation from G to A at the 21^st^ nucleotide compared with the reference gga-miR-1579. In total, isomiRs of 116 known chicken miRNAs, including 82 in tumorous spleen, 69 in MD lymphoma, 70 in non-infected spleen, and 82 in non-infected lymphocytes, had more counts than reference miRNA sequences listed in miRBase 16.0. Details of isomiRs for each known miRNA are presented in Table S2, with sequences perfectly matched with reference pre-miRNAs listed at the top, followed by one-error alignments.

In addition, we characterized the counts of 46 mature miRNAs derived from the two arms of 23 precursor miRNAs (Table 3). Of them, 15 miRNAs originating from the 5′ (5p) ends had more counts than those originating from 3′ (3p) ends. Three 3p-derived miRNAs (gga-miR-199-3p, gga-miR-22-3p, and gga-miR-140-3p) had more counts than their corresponding 5p-derived miRNAs. Four precursor miRNAs (gga-mir-126, gga-mir-1728, gga-mir-301b, and gga-mir-455) showed a reversal in the ratios of the 5p- and 3p- derived sequences across the four RNA libraries.

**Table 3:** Counts of known miRNAs originating from 5′ or 3′ arm of precursors in four libraries.

		MDV-infected				Non-infected			
		Tumorous spleen		Lymphoma(liver tumor)		Spleen		Lymphocytes(peripheral blood)	
Index	Pre-miRNA	5p	3p	5p	3p	5p	3p	5p	3p
1	gga-mir-181a	6294^a^	271	4725	78	23970	238	55572	1297
2	gga-mir-142	9417	2495	5752	866	9915	610	38470	15528
3	gga-mir-10a	7733	90	869	4	8317	271	416	-
4	gga-mir-146b	11348	85	25346	57	4703	14	15407	65
5	gga-mir-99a	1876	37	955	41	3359	78	17989	298
6	gga-mir-146c	31407	594	99065	721	2432	25	16804	193
7^b^	gga-mir-126	612	906	110	139	1349	1095	671	337
8	gga-mir-17	877	398	493	280	1133	446	3396	1432
9	gga-mir-1456	224	7	80	–	106	–	606	5
10	gga-mir-1729	55	–	11	–	105	10	381	102
11	gga-mir-15c	401	4	123	–	102	–	2283	27
12	gga-mir-1329	42	5	42	–	23	–	289	13
13	gga-mir-1552	21	5	3	–	10	6	95	24
14	gga-mir-1551	20	3	17	3	8	–	38	4
15	gga-mir-1451	20	16	11	–	6	–	61	47
16	gga-mir-1736	–	–	–	–	–	–	3	3
17^b^	gga-mir-1728	–	–	4	–	–	3	18	6
18^b^	gga-mir-301b	27	18	20	3	3	24	245	121
19^c^	gga-mir-199	284	61748	64	13939	948	243757	74	23853
20^c^	gga-mir-22	96	1818	19	497	87	1755	238	5951
21^c^	gga-mir-140	43	45110	14	24393	57	52749	277	220818
22^b^	gga-mir-455	48	65	6	3	20	45	-	-
23	gga-mir-30a	4994	381	2736	85	7650	675	7297	561

a. Numbers indicate counts for all isomiRs originating from 5**′**(5p) or 3**′**(3p) arms of the miRNA precursor.

b. miRNAs from two arms showed a reversal in the ratios across four RNA libraries.

c. miRNAs deriving from 3′ ends of precursor had more counts than that from 5′ ends.

### Novel miRNA Candidate Identification

There were 68, 155, and 703 chicken miRNA candidates in subgroup1, 2, and 3, respectively (Table S3). One MDV miRNA candidates in each of subgroup1 and subgroup3 was identified. In the present study, 42 of 68 novel miRNA candidates in subgroup1 were new 3p-derived sequences, and 26 were new 5p-derived sequences. Of the 68 candidates (Table S3), 40 miRNA candidates were mapped to other vertebrate known mature miRNAs and 41 were detected previously by Glazov et al. [24]. As expected, the counts of known mature miRNAs were usually much higher than their corresponding novel miRNA* (star) candidates, and only eight novel candidates showed higher counts than the annotated known mature miRNAs in at least one library (Table S4). The results are consistent with the known mechanisms of miRNA biogenesis and strand preference. A small RNA duplex is generated from a hairpin-like miRNA precursor. The strand with lower thermodynamic stability at its 5′ end is more easily incorporated into the RISC complex, and the other inactive strand, called miRNA* (star) is rapidly degraded [27]. Usually, miRNA* cannot be detected by conventional methods due to their rapid turnover; however, the high sensitivity of deep sequencing allows their identification.

### Verification of Novel miRNAs

To further verify novel miRNAs in the four samples, we produced customized miRNA arrays including 533 novel miRNA candidate probes (Table S5). In total, 276 out of 533 probes were detectable. There were 18, 74, and 184 miRNA candidates in subgroup1, 2, and 3, respectively, detected in microarrays. The microarray signals and Solexa deep sequencing counts for the 276 detectable probes are listed in Table S6. Of the 276 detectable probes, 17 novel miRNA candidates with counts ranged from 1 to 11374 (Table S7), including 11 candidates from subgroup2, and six from subgroup3 were further verified by qPCR and amplicons were cloned into pMD-19T plasmid and sequenced (Figure S1).

### Differential Expression Analysis of Known miRNAs between MDV-infected and Non-infected Samples

**Figure 2:**
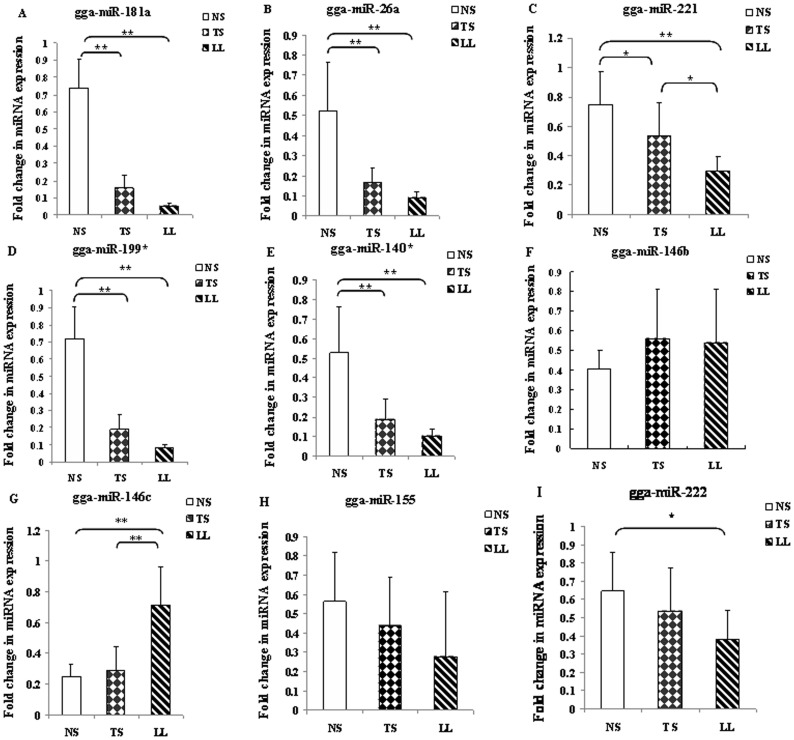
Expression of nine chicken known miRNAs. A. Expression of gga-miR-181a among TS, LL, and NS. B. Expression of gga-miR-26a among TS, LL, and NS. C. Expression of gga-miR-221 among TS, LL, and NS. D. Expression of gga-miR-222 among TS, LL, and NS. E. The expression of gga-miR-199* among TS, LL, and NS. F. Expression of gga-miR-140* among TS, LL, and NS. G. The expression of gga-miR-155 among TS, LL, and NS. H. Expression of gga-miR-146b. I. Expression of gga-miR-146c among TS, LL, and NS. TS: MDV-infected tumorous spleen; LL: MD lymphoma from liver; NS: non-infected spleen.

Differential expression of miRNAs between MDV-infected and non-infected samples based on counts was conducted using a web tool, IDEG6 [28]. Because the perfect-match isomiRs might not be the unique functional isoform [29], counts from different isoforms of the same miRNA were pooled. Non-infected spleen was used as the control to identify differentially expressed miRNAs in tumorous spleen (Table S8). Because the liver tumor was a lymphocytic solid tumor, consisting of a heterogeneious mixture of oncogenic, inflammatory, and immunologically active and inactive cells [30], both the non-infected spleen and non-infected lymphocytes were used as controls to identify differentially expressed miRNAs in the MD lymphoma from liver (Table S9). There were 94 and 63 miRNAs deregulated in tumorous spleen and MD lymphoma compared with their corresponding controls. The differentially expressed miRNAs that occurred in both tumorous spleen and MD lymphoma from liver were considered as deregulated miRNAs in MDV-infected samples during tumor transformation. In total, 11 up-regulated and 28 down-regulated miRNAs in MDV-infected samples were identified (Table S10). Of these 39 miRNAs, four down-regulated miRNAs (gga-miR-221, −140*, −199*, and 181a) and four up-regulated miRNAs (gga-miR-146c, −146b, −222, and −155) in the two MDV-infected samples with high counts were selected to be further verified by qPCR among MDV-infected whole spleens with tumors (tumorous spleens), MD lymphomas from liver, and non-infected spleens. Additionally, because of previous reports on gga-miR-26a [18], [21], [23], it was also chosen to be verified by qPCR, which showed down-regulation in MD lymphoma from liver compared with non-infected lymphocytes and non-infected spleen in the current study (Table S9). The differential expression profiles of seven miRNAs (gga-miR-221, −140*,−199*, 181a, −146b, −146c, and −26a) were consistent between IDEG6 analysis and qPCR. gga-miR-181a, gga-miR-26a, gga-miR-221, gga-miR-199*, and gga-miR-140* were down-regulated (Figure 2A–E). gga-miR-146b showed a trend toward being higher, and gga-miR-146c was up-regulated in tumorous spleen and MD lymphoma compared to non-infected spleens (Figure 2F, G). Only two miRNAs (gga-miR-155 and −222) showed different results from the two methods. gga-miR-155 was up-regulated in two MDV-infected samples in IDEG6 analysis, but it showed a trend toward down-regulation (*p*>0.05) in MDV-infected compared to non-infected samples by qPCR (Figure 2H). gga-miR-222 was up-regulated in MDV-infected samples in IDEG6 analysis; however, it was down-regulated in MD lymphoma from liver (not MDV-infected spleen) compared to non-infected spleen by qPCR (Figure 2I).

### Prediction of Potential Targets for Nine Known miRNAs

**Figure 3:**
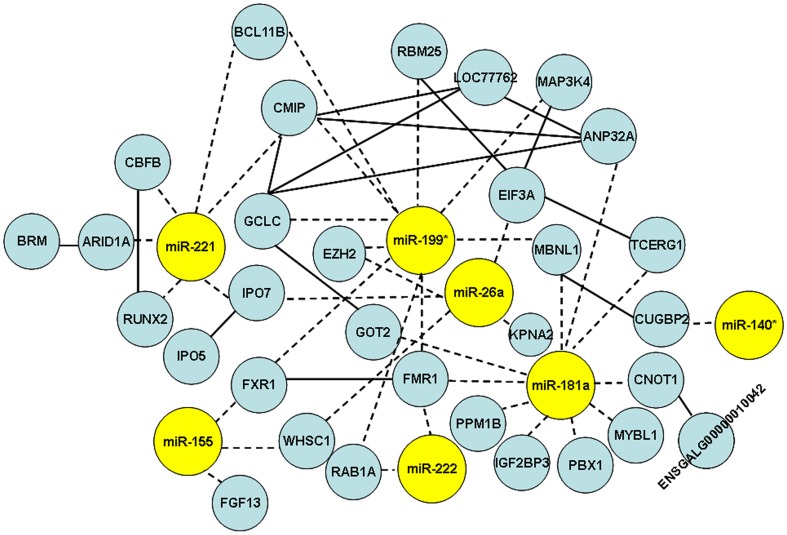
Network of differentially expressed miRNAs and their putative targets. In the network, miRNAs are represented by yellow circles and targets are represented by blue nodes. Solid lines show target-target interactions; dashed lines show miRNA-target interactions.

Putative target genes of gga-miR-181a, gga-miR-26a, gga-miR-221, gga-miR-222, gga-miR-155, gga-miR-146b, and gga-miR-146c were predicted by TargetScan (Version 6.0) [31]. Target genes of gga-miR-199* and gga-miR-140*, for which whose seed sequences could not be found in TargetScan, were predicted by TargetScanHuman Custom (Version 5.3). In total, 1598 annotated mRNA transcripts were predicted (Table S11). Because negatively correlated expression patterns between miRNAs and their corresponding targets have been reported [32], we used differentially expressed genes in tumorous spleens versus non-infected spleens from our previous microarry study on gene expression [33] to narrow down candidate targets. The genes presenting inverse expression patterns compared to their corresponding miRNAs were selected. In total, 189 candidate targets which were deregulated in tumorous spleens in the previous study were identified (Table S12). To investigate interactions of miRNAs and targets, and also interaction of targets and targets, the protein-protein interactions from STRING were extracted, and network was drawn (Figure 3). Only one target gene for miR-26a, *EZH2* was reported previously [34]. All other interactions of miRNAs and targets were proposed for the first time in this study, and can now be considered as candidates for further research.

### Target Validation using Luciferase Reporter Gene Assays

**Figure 4:**
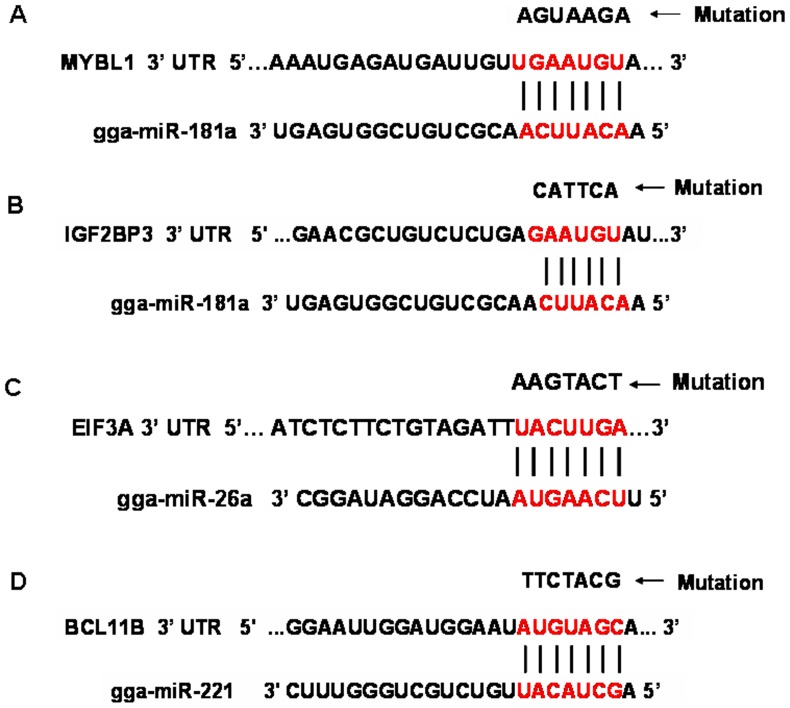
Schema of miRNA binding sites in corresponding 3′-UTR sequence of chicken predicated target gene (seed sequence highlighted in red). A. Schema of binding site for gga-miR-181a and MYBL1; B. Schema of binding site for gga-miR-181a and IGF2BP3; C. Schema of binding site for gga-miR-26a and EIF3A; D. Schema of binding site for gga-miR-221 and BCL11B. Arrow points to mutated target gene 3′-UTR eliminating the seed binding site.

**Figure 5:**
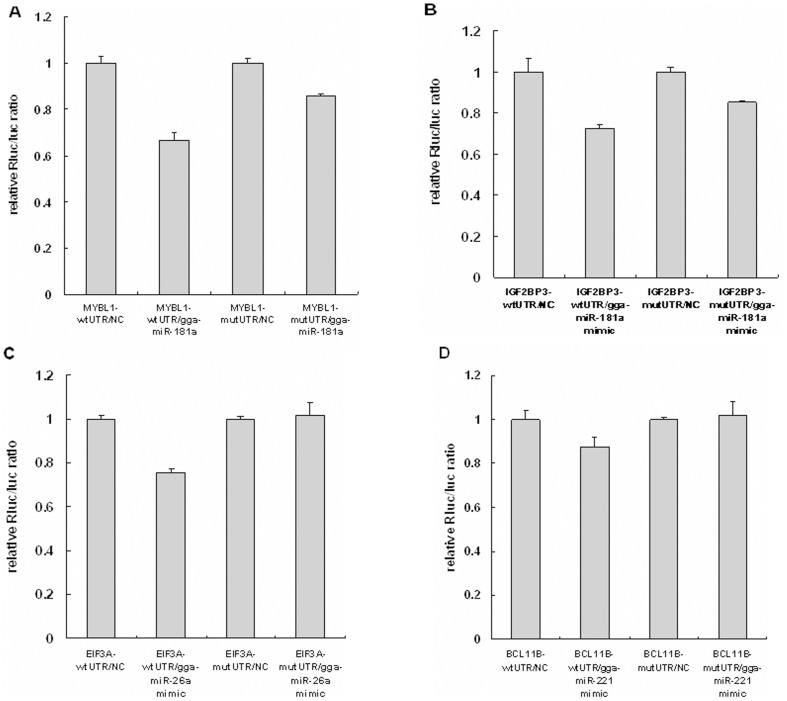
Target validation using luciferase reporter assays. A. Interaction validation for gga-miR-181a and MYBL1. B. Interaction validation for gga-miR-181a and IGF2BP3. C. Interaction validation for gga-miR-26a and EIF3A. D. Interaction validation for gga-miR-221 and BCL11B. 293T cells were co-transfected with pmiR-RB-REPOR-UTR, or pmiR-RB-REPOR-mutated UTR and miRNA mimic, or negative control. Gene-wtUTR: wild type 3′UTR of predicted target gene. Gene-mutUTR: Mutated 3′UTR of predicted target gene. NC: Negative control.

To further verify the interaction of predicted target genes and miRNAs, we conducted luciferase reporter gene assays to detect interaction of gga-miR-181a with *MYBL1* and *IGF2BP3*, gga-miR-26a with *EIF3A*, and gga-miR-221 with *BCL11B*. The pmiR-RB-REPORT-UTR and pmiR-RB-REPORT-mutated UTR were transfected into 293 T cells with miRNA mimic or negative control (Figure 4). The luciferase activity was significantly reduced, by 33%, when a gga-miR-181a mimic was co-transfected with pmiR-RB-REPORT-MYBL1UTR into 293T cells, suggesting that significant interaction occurs between them (Figure 5A). Transfection of the gga-miR-181a mimic also inhibited luciferase activity of pmiR-RB-REPORT-IGF2BP3 UTR by 27% (Figure 5B). The gga-miR-26a mimic inhibited luciferase activity of pmiR-RB-REPORT-EIF3A UTR by 24% (Figure 5C). The gga-miR-221 mimic slightly decreased luciferase activity of pmiR-RB-REPORT-BCL11B UTR by 12% (Figure 5D). These results demonstrate significant interaction of gga-miR-181a with *MYBL1* and *IGF2BP3*, and of gga-miR-26a with *EIF3A*.

## Discussion

In this study, two MDV-infected samples (tumorous spleen and MD lymphoma from liver) were selected for analysis. They represented two types of visceral tumors that frequently occur after MDV infection. Typically, very tiny tumor nodules appeared throughout the spleen in the current study and they could not be reliably dissected free from adjacent splenic tissue; therefore, whole spleens including the tumors (termed, tumorous spleens) were harvested for analysis. However, MDV-induced tumors in the liver were large and mature tumors and, thus, could be dissected free from adjacent non-tumor tissue and harvested. Generally, MD tumors are characterized by lymphocyte infiltration. Lymphomatous lesions in visceral organs are proliferative, consisting of proliferating small to medium lymphocytes, lymphoblasts, and activated and primitive reticulum cells [30]. In MD lymphomas from liver, although residual liver cell-derived miRNAs may contribute to the results, the contribution of hepatocytes is expected to be far less than that of the much greater numbers of infiltrating lymphocytes. Considering the cellular and lymphocytic properties of the selected MDV-induced tumor tissues, non-infected whole spleen and lymphocytes from peripheral blood were chosen as the two controls in deep sequencing.

We found that MDV-infected samples had higher percentages than non-infected samples in mRNA, RFam, and repbase categories, which indicates that these known RNA species were activated during MDV infection and might play important roles in MDV-related pathology. In addition, isomiRs were very common, and many isomiRs had more counts than the corresponding miRNA sequences listed in miRBase. They could, therefore, be considered for use as reference sequence under pathological conditions of MDV infection. In addition, differential expression of miRNA counts was analyzed by IDEG6. Because of the lack of biological replicates and technical duplicates in sequencing, a very stringent p-value cut-off (*P*<0.001 simultaneously in Audic-Claverie test, Fisher exact test, and Chi-squared 2×2 test with the Bonferroni correction to adjust for pair-wise comparison) was applied to identify differentially expressed miRNAs [35], [36]. Moreover, differentially expressed miRNAs occurring in both of the two comparison groups, (MDV-infected tumorous spleen vs. non-infected spleen, and MD lymphoma vs. non-infected spleen/lymphocytes) were considered to be deregulated miRNAs in MD tumors compared to non-infected controls. Only 11 up-regulated and 28 down-regulated miRNAs were identified. The relatively small number of deregulated miRNAs might result from the conservative statistical thresholds used in differential expression analysis. Subsequently, differential expression of nine miRNAs was further investigated by qPCR, and seven were verified.

In the current study, miR-181a and miR-26a were down-regulated in tumorous spleen and MD lymphoma, compared to non-infected spleen. Several previous studies provide evidence of their involvement in cancer. Two members of miR-181 (miR-181a, 181b) were down-regulated in glioma specimens and glioma cell lines [37], as well as pituitary adenomas [38] and lymphocytic leukemia [39]. It has been suggested that suppression of miRNA-181a may increase cell growth in the lung carcinoma cell line, A549 [40]. The expression of miR-181a,b was also decreased in avian influenza virus (AIV)-infected chicken lungs and tracheae, and they were proposed as strong miRNA candidates for regulating host response to AIV infection [41]. Acting as a tumor suppressor, miR-181a triggered growth inhibition, induced apoptosis, and blocked invasion in glioma cells. Deregulation of miR-181a may be a critical factor for malignancy occurrence in human gliomas [42]. The luciferase reporter gene assay indicated significant interaction of miR-181a with two predicted target genes, *MYBL1* and *IGF2BP3*. *MYBL1* acting as an oncogene, was associated with tumorigenesis [43], and its expression was elevated in human cancers [44], [45]. *IGF2BP3* is considered to be the first biomarker of prognostic significance in ovarian clear cell carcinoma [46]. Its expression has been observed in many malignant neoplasms, including cervical carcinomas [47] and pancreatic ductal adenocarcinoma tissues [48]. The gene expression of *MYBL1* and *IGF2BP3* was up-regulated in tumorous spleen in our previous microarray study [33]. Their up-regulation in tumorous spleen might result from down-regulation of miR-181a.

miR-26a is considered to be a potential tumor suppressor [49]. Suppression of miR-26a has been demonstrated in various human cancers, such as thyroid anaplastic carcinoma [50] and Burkitt’s lymphoma [34], [50]–[52]. A recent miRNA microarray study has shown that miR-26a was down-regulated in nasopharyngeal carcinoma (NPC) tissues compared with adjacent normal tissues [53]. Chen et al. showed that miR-26a inhibited cell growth and colony formation, induced a G1 arrest in NPC cells, and suppressed tumorigenesis in a murine model of NPC xenograft [53]. In addition, miR-26a was also down-regulated in seven independently-derived MDV-transformed lymphoblastoid T cell lines, and ALV and REV transformed lymphoid cell lines [21]. As the only known target gene of miR-26a, *EZH2*, acting as an oncogene, was shown to be involved in proliferation of different cell types, including tumor cell lines [54]; it showed oncogenic activity and tumor transformation requiring histone methylatransferase activity. *EZH2* has been detected in a variety of neoplastic cells of poorly differentiated invasive carcinomas [55]. Its catalytic activity was shown to be essential for oncogenic transformation [54]. *EIF3A*, another predicted target gene of miR-26a was up-regulated in tumorous spleen of our previous study [33]. Overexpression of *EIF3A* occurs in many solid tumors and cancer cell lines including human breast [56], cervical [57], esophageal [58], lung [59], gastric cancers [58] and Hela cells [59]. It is regarded as prognostic biomarker for poor clinical outcome [60]. Significant interaction of miR-26a and *EIF3A* was verified in the current study by using the luciferase reporter gene assay, which indicated that down-regulation of miR-26a could induce expression of *EIF3A* in MD lymphoma.

miR-221 and miR-222 are considered to be potentially oncogenic miRNAs. They are involved in tumorigenesis and affect the expression of cell cycle regulatory proteins in different human cancers [61], [62]. The regulation of miR-221 in different cancer types appears to be variable. Both miR-221 and miR-222 were over-expressed in tumors of the lung, breast, pancreas and in glioblastoma, and papillary thyroid carcinoma, [37], [63]–[66]. However, their expression in prostate cancer and chronic lymphocytic leukemia was down-regulated [1], [10], [67]. The same discrepancy has been reported in MDV-transformed samples. Up-regulation of miR-221 and miR-222 was observed in MSB1 [22], [23], [61]. However, the increased expression was unique to the MSB1 cell line, while in some other MDV transformed cell lines, such as MDCC-226S (T226S) and MDCC-265L (T265L), levels of miR-221 and miR-222 were unchanged or down-regulated [61]. The results may be due to dissimilarities among these cell lines, with MSB1 being infected by BC1, while T226S and T265 are derived from a lymphoma infected by RB1B, which is more lymphomagenic than BC1 [61]. In the current study, expression of miR-221 and miR-222 was down-regulated in MDV-infected samples, especially in MD lymphoma, which was consistent with a previous report that miR-221 was slightly down-regulated in MDV-induced splenic tumors [18].

Differential expression of miR-199* and miR-140* in MDV-infected samples was reported here for the first time. Both were down-regulated in tumorous spleens and MD lymphoma. To our knowledge, this is the first report of involvement of miR-140* in tumors. Its significant down-regulation in the current study strongly implicates involvement of miR-140* in oncogenic transformation. There are limited reports about the function of miR-199* in cancer. Murakami et al. reported that miR-199 and miR-199* were down-regulated in hepatocellular carcinoma compared with adjacent non-tumorous tissue [68]. The down-regulation of miR-199* in MDV-infected samples indicates its role in tumorigenesis.

Some predicted target genes for miR-221, 222, and 199*, such as *RUNX2*, *BCL11B*, and *RAB1A*, show oncogenic characteristics in tumors. *BCL11B*, as a predicted target for miR-221 and miR-199*, has been implicated in T-cell development, differentiation, and proliferation [69]. Its overexpression was detected primarily in T-cell malignancies, which was a characteristic feature of T-cell acute lymphoblastic leukemia (T-ALL) [70]. Our previous microarray results showed it was up-regulated in tumorous spleen [33]. However, the luciferase reporter gene assay showed only slight interaction between miR-221 and *BCL11B.* Although *BCL11B* is likely not the target gene of miR-221, its involvement in tumorigenesis is still suggested because of its up-regulation in MD tumors.

The differential expression of miR-146b and miR-146c between MDV-infected and non-infected samples was analyzed. In the current study, miR-146b showed an increasing trend, and miR-146c was significantly up-regulated in tumorous spleens and MD lymphomas compared to non-infected spleens. In humans, elevated expression of miR-146b was observed in malignant myoepithelioma of breast [71] as well as papillary thyroid carcinoma [63], [72]. Nikiforova et al. reported that miR-146b was overexpressed at least 2-fold in papillary thyroid cancer compared with benign hyperplastic nodules [73]. In our study, miR-146b only showed an increasing trend, whereas its isoform, miR-146c, which has been only reported in chicken and zebra finch to date, showed significantly elevated expression level in tumorous spleen and MD lymphoma. Although the specific function of miR-146c and its predicted target gene are unknown, the elevated expression of miR-146c in MDV-induced tumor indicates its important role in lymphomagenesis.

### Conclusion

Solexa deep sequencing is an effective high-throughput approach to investigate the miRNA repertoire. In this study, 187 known chicken and 16 MDV miRNAs were identified, and 17 novel miRNA candidates were confirmed by qPCR. Abundant isomiRs were detected; therefore, they can be utilized to refine the miRNA annotation in miRBase. Six miRNAs (gga-miR-181a, gga-miR-26a, gga-miR-221, gga-miR-222, gga-miR-199*, and gga-miR-140*) were down-regulated and one miRNA (miR-146c) was up-regulated in MDV-infected samples, and especially in MD lymphomas, compared to non-infected samples. Interactions of miRNAs and predicted target genes, including gga-miR-181a with *MYBL1* and *IGF2BP3*, and gga-miR-26a with *EIF3A,* were discovered by using the luciferase reporter gene assay. Collectively, differential expression of miRNAs and target genes in MD lymphoma strongly suggests that they are involved in MDV-induced lymphoma transformation and they facilitate neoplastic transformation.

## Materials and Methods

### Sample Collection

Chickens from a White Leghorn specific pathogen free line (BWEL) [74] were used. One hundred randomly selected chickens were infected intraperitoneally at one day of age with 2000 PFU of the MDV GA strain (passage 9) and the remaining 50 chickens were injected with the same dosage of diluent (0.2 mL) as controls. The two groups were kept in separate isolators in different rooms. The trial period lasted to 56 days postinfection (d.p.i.). During this phase, the chickens’ clinical signs were observed 2–3 times daily and severely morbid birds were euthanized. Whole spleens with small tumors were removed, and MDV-induced tumors in liver were dissected from adjacent non-tumor tissue and harvested. Whole spleens and lymphocytes from peripheral blood were harvested from age- and sex-matched non-infected birds, to serve as sources of control tissue. Animal experiments were approved by the Animal Care and Use Committee of China Agricultural University (Approval ID: XXCB-20090209) and the experiment was performed according to regulations and guidelines established by this committee. All tissues and lymphocytes were immediately stored in RNAfixer (BioTeke Co., Ltd, Beijing, China) at 4°C overnight and then transferred to −80°C until RNA isolation. Four samples (MDV-infected whole spleen, MD liver lymphoma, non-infected spleen, and non-infected lymphocytes) were used to perform high-throughput Solexa deep sequencing. The whole spleen with tumors and the MD lymphoma from liver were from the same MDV-infected chicken at 46 d.p.i.; the non-infected spleen was collected at 40 d.p.i., and the lymphocytes were from two chickens at 40 d.p.i. In addition, eight tumorous spleens (whole spleens including tumor and adjacent non-tumor tissue), eight MD liver lymphomas (dissected from adjacent non-tumor tissue), and eight non-infected whole spleens were harvested to detect differential expression of miRNAs (Table 4).

**Table 4:** Samples from three groups used in qPCR.

MDV-infected spleens		MD lymphomas from livers		Non-infected spleens	
Sex	Days post infection	Sex	Days post infection	Sex	Days post infection
Female	35	Female	31	Female	33
Female	36	Female	36	Female	38
Female	37	Female	37	Female	40
Female	38	Female	38	Female	42
Male	38	Male	40	Male	40
Male	46	Male	46	Male	40
Male	48	Male	48	Male	48
Male	55	Male	55	Male	55

### Total RNA Isolation, Small RNA Library Construction and Deep Sequencing

Total RNA of the individual four samples was extracted using the mirVana miRNA Isolation Kit (Ambion, Austin, USA) according to the manufacturer’s protocol. Briefly, 10 µg of RNA sample was size fractionated with a YM-100 column (Millipore). The larger RNA fraction (>100 KDa) was removed. The smaller RNA fraction (<100KDa) was further fractioned on a 15% tris-borate-EDTA (TBE) urea polyacrylamide gel and the 15–50 nt fraction was obtained. Subsequently, 5′ and 3′ RNA adapters were ligated to the small RNA by T4 RNA ligase (Promega), followed by reverse transcription and PCR product purification. Purified products were quantified on the TBS-380 mini-fluorometer (Turner Biosystems) using Picogreen® dsDNA quantitation reagent (Invitrogen), then diluted to 10 nM and transferred for sequencing on the Illumina/Solexa G1 sequencer (Illumina, San Diego, USA ). Deep sequencing data has been deposited in NCBI’s Gene expression Omnibus (GEO). The accession numbers are: Series, GSE31349; Samples, GSM777474-GSM777477.

### Data Processing

Data processing followed the procedures as described in a previous study [75]. Briefly, the raw reads were subjected to the Illumina pipeline filter (Solexa 0.3), and then the dataset was further processed with an in-house program, ACGT101-miR (LC Sciences, Houston, Texas, USA) [75]–[77] to remove adapter dimers, junk, low complexity, low copy (Copy # <3) reads, common RNA families (mRNA, rRNA, tRNA, snRNA, snoRNA) and repeats. Subsequently, unique sequences were mapped to chicken or MDV precursors in miRBase 16.0 by BLASTn search to identify known miRNAs and novel 3p- and 5p- derived miRNAs. Length variation at both 3′ and 5′ ends and one mismatch inside of the sequence were allowed in the alignment. The unique sequences mapping to chicken or MDV mature miRNAs in hairpin arms were identified as known miRNAs. The unique sequences mapping to the other arm of known chicken or MDV precursor hairpin opposite to the annotated mature miRNA-containing arm were considered to be novel 5p- or 3p-derived miRNA candidates. The remaining sequences were mapped to other vertebrate or herpesvirus precursors (with the exclusion of chicken and MDV) in miRBase 16.0 by BLASTn search, and the mapped pre-miRNAs were further BLASTed against the chicken and MDV genomes to determine their genomic locations. The unmapped sequences were BLASTed against the chicken and MDV genomes, and the hairpin RNA structures containing sequences were predicated from the flank 60 nt sequences using RNAfold software (http://rna.tbi.univie.ac.at/cgi-bin/RNAfold.cgi). The criteria for secondary structure prediction [73] were: (1) number of base pairs in a stem ≥16 bp; (2) number of allowed errors ≤18; (3) free energy (DG) ≤ −15 kCal mol-1; (4) percentage of miRNA appearing in the stem ≥80%; (5) length of hairpin≥53 nts; and (6) length of hairpin loop≤22 nts.

### Microarray Analysis

To confirm results of deep sequencing, we performed microarray analysis with customized probes, which were produced by a service provider (LC Sciences, Houston, USA). Briefly, total RNA of the four samples used for sequencing was extracted using a mirVana Isolation kit (Ambion, Austin, USA) and 5 µg total RNA was fractioned with a YM-100 Microcon centrifugal filter (Millipore, Billerica, MA, USA). Small RNAs (<300 nt) were 3′-extended with a poly (A) tail by poly (A) polymerase and then Cy5 was ligated to the poly-A tails. In total, there were 533 customized probes to detect novel miRNA candidate sequences; in addition, 28 sequences, including endogenous 5S rRNAs and artificially synthesized single base mismatch probes, were added as positive and negative controls, respectively. Each detection probe was run in triplicate. Hybridization was carried out on a *µ*Paraflo™ microfluidic chip with 100 µL 6xSSPE buffer (0.90 M NaCl, 60 mM Na_2_HPO_4_, 6 mM EDTA, pH 6.8) at 34°C overnight using a micro-circulation pump (Atactic Technologies, Houston, TX, USA). Hybridization images were collected by using a laser scanner (GenePix 4000B, Molecular Device, Sunnyvale, CA, USA) and digitized by Array-Pro image analysis software (Media Cybernetics, Bethesda, MD, USA). The signal values were derived by background subtraction and normalization. A transcript must have met at least three criteria to be considered detectable: signal intensity >3x (background standard deviation), spot CV <0.5 (CV was calculated by standard deviation/signal intensity), and at least 2 of 3 replicate probes were above detection level.

### Analysis of Differentially Expressed miRNAs Based on Counts in Deep Sequencing

miRNA differential expression based on deep-sequencing counts was analyzed using a web tool, IDEG6 [28], http://telethon.bio.unipd.it/bioinfo/IDEG6_form/. The Audic and Claverie, Fisher exact test, and Chi-squared 2×2 test were all conducted, and the significance threshold was set to be 0.001 in each test. Bonferroni correction was applied as a correction for multiple testing.

### qPCR for Novel miRNA Candidate Verification and miRNA Differential Expression

In total, confirmation of 17 novel miRNA candidates in tumorous spleen and differential expression of nine known chicken miRNAs in 24 samples was performed by using a stem-loop RT-PCR. The stem-loop RT primer, and regular forward and reverse primers were designed (Table S13). Total RNA was isolated by using the mirVana miRNA Isolation Kit (Ambion, Austin, USA). Reverse transcription was performed in 25 µL total volume, including RNA, 1 × RT buffer, 50 nM stem-loop RT primer, 0.25 mM each dNTPs, 3.33 U/ml MultiScribe reverse transcriptase and 0.25 U/ml RNase inhibitor. The optimum thermal cycling parameters were 16°C for 30 min, 42°C for 30 min, 85°C for 5 min. Real-time PCR reactions were performed in the ABI 7300 system (Applied Biosystems, Foster City, Calif., USA) in a final volume of 15µL with 1µL of RT production, 1.5 mM forward primer, 0.7 mM reverse primer, and 1×PCR mix (Power SYBR® Green PCR Master Mix, Applied Biosystems, US). The optimum thermal cycling parameters included 95°C for 10 min, 40 cycles of 95°C for 15 s, 60°C for 1 min, 95°C for 15 s, 60°C for 30 s, and 95°C for 15 s. Each individual sample and no-template control was run in triplicate, and the average critical threshold cycle (Ct) was calculated. The PCR products were cloned by using pMD-19T plasmid and DH5α and sequenced to ensure that the correct miRNA were amplified. For differential expression of nine known miRNA, 28 s was used as the internal reference gene [14]. The 2^−ΔΔCt^ method was used to calculate relative gene expression levels across the three groups. All analyses were performed using GLM model of SAS (SAS Institute, Cary, NC).

### Validation for Interaction of miRNAs and Predicted Target Genes using Luciferase Reporter Gene Assays

A pmiR-RB-REPORT™ vector [78] (Ribobio Co., Guangzhou, China) was used for 3′ UTR-luciferase reporter assays to detect interactions of gga-miR-181a with *MYBL1* and *IGF2BP3*, gga-miR-26a with *EIF3A*, gga-miR-221 with *BCL11B*. The TargetScan Human database 6.1 http://targetscan.org/was used to identify miRNA binding sites. The gga-miR-181a mimic, gga-miR-26a mimic, gga-miR-221 mimic and their corresponding negative controls (Ribobio Co.) were transfected with pmiR-RB-REPORT™-target gene UTR or pmiR-RB-REPORT™-target gene mutated UTR. Reporter assays were conducted in triplicate using 293T cells. Transfection was done according to the manufacturer’s instructions for Lipofectamine™ 2000 (Invitrogen, Carlsbad, CA) with 50 nM mimic and 100 ng/mL reporter plasmid.

## Supporting Information

Figure S1 **Results for novel miRNA candidate sequencing.** Novel miRNA candidates were verified by qPCR in tumorous spleens and amplicons were cloned and sequencing. A–Q shows sequences of PN-cfa-miR-107_R+2, PN-xtr-miR-23a_R+1, PN-xtr-miR-20b_R+1, PN-xtr-miR-214_R-4, PN-xtr-miR-26_R+3, PN-tgu-miR-425-5p_R+1, PN-tgu-miR-1388, PN-rno-miR-214_R+1, PN-xtr-miR-125b_L-1R+1, PN-hsa-miR-150_12TC, PN-mmu-miR-1937a_L-1_18CT, PC597-5p, PC556-3p, PC30-5p, PC495-3p, PC34-3p, and PC306-5p, respectively. Sequences in red frames are miRNA candidates. Sequences in blue frames are reverse complementary sequences of novel miRNA candidates.
(TIF)

Table S1 **Known chicken and MDV miRNAs detected in four samples.**
(XLS)

Table S2 **isomiRs for known chicken miRNAs in four samples.**
(XLS)

Table S3 **Novel chicken and MDV miRNA candidates from subgroup1, 2, and 3 in four samples.**
(XLS)

Table S4 **Counts for novel 3′ or 5′-derived miRNA candidates and their corresponding known miRNAs in four samples.**
(XLS)

Table S5 **Novel miRNA candidate probes.**
(XLS)

Table S6 **Microarray signals and deep sequencing counts for 276 probes.**
(XLS)

Table S7 **Seventeen miRNAs verified by qPCR.**
(XLS)

Table S8 **Differentially expressed miRNAs in MDV-infected tumorous spleen analyzed by IDEG6.**
(XLS)

Table S9 **Differentially expressed miRNAs in MD lymphoma analyzed by IDEG6.**
(XLS)

Table S10 **Differential expression analysis of miRNAs in MDV-infected compared with non-infected samples using IDEG6.**
(XLS)

Table S11 **Predicted targets of nine known chicken miRNAs.**
(XLS)

Table S12 **Candidate target genes of miRNAs.**
(XLS)

Table S13 **Primers used in qPCR for seventeen miRNA candidates and nine known miRNAs.**
(XLS)
