# Supplementary figures and images for: A Systematic Analysis of miRNA Transcriptome in Marek’s Disease Virus-Induced Lymphoma Reveals Novel and Differentially Expressed miRNAs
Source: PLoS One. 2012 Nov 30;7(11):e51003. doi: 10.1371/journal.pone.0051003 (PMC3511444; doi:10.1371/journal.pone.0051003)

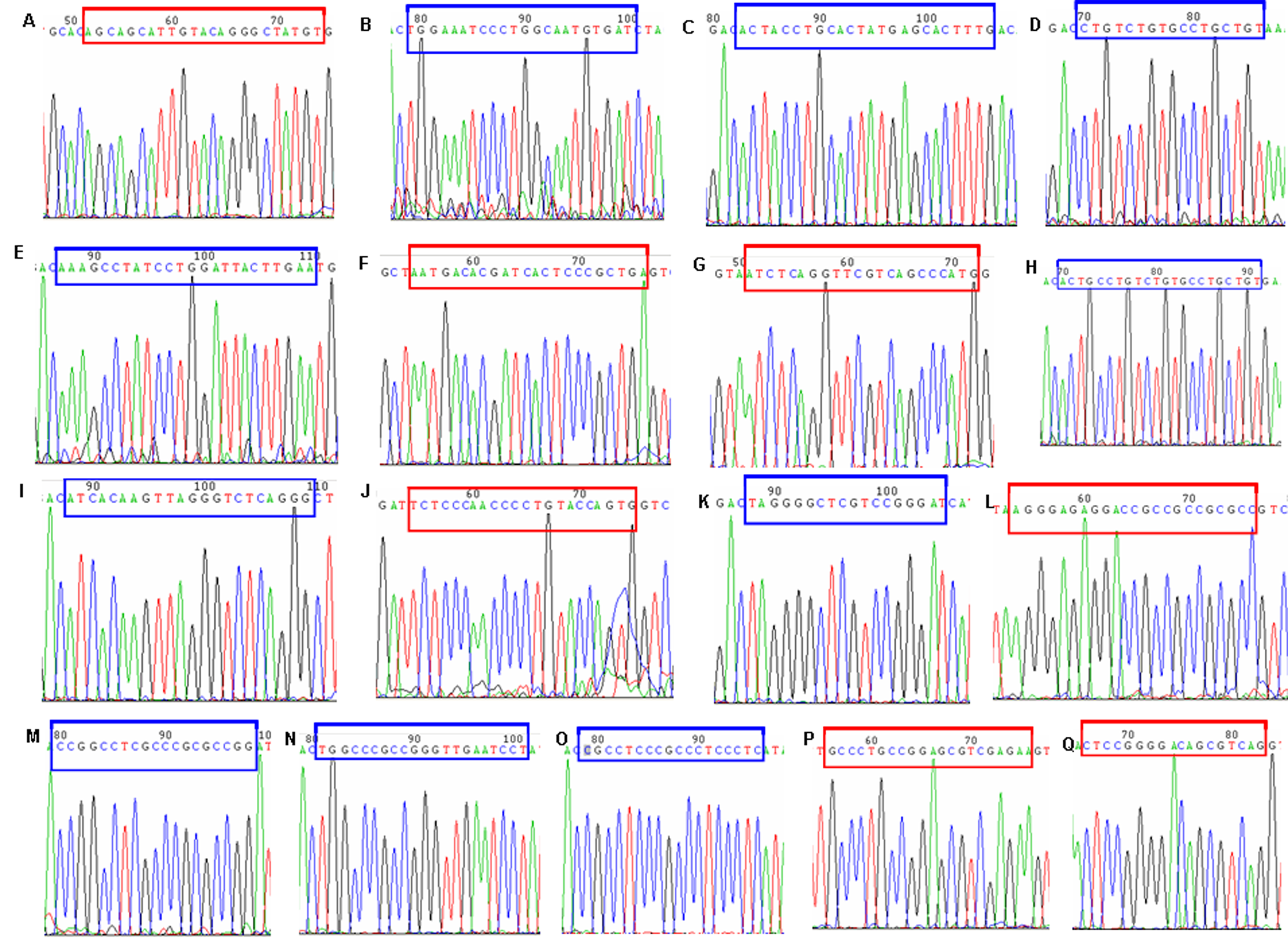

Supplement: Figure S1 — Results for novel miRNA candidate sequencing. Novel miRNA candidates were verified by qPCR in tumorous spleens and amplicons were cloned and sequencing. A–Q shows sequences of PN-cfa-miR-107_R+2, PN-xtr-miR-23a_R+1, PN-xtr-miR-20b_R+1, PN-xtr-miR-214_R-4, PN-xtr-miR-26_R+3, PN-tgu-miR-425-5p_R+1, PN-tgu-miR-1388, PN-rno-miR-214_R+1, PN-xtr-miR-125b_L-1R+1, PN-hsa-miR-150_12TC, PN-mmu-miR-1937a_L-1_18CT, PC597-5p, PC556-3p, PC30-5p, PC495-3p, PC34-3p, and PC306-5p, respectively. Sequences in red frames are miRNA candidates. Sequences in blue frames are reverse complementary sequences of novel miRNA candidates. (TIF) [file pone.0051003.s001.tif]
